# Win ratio analysis of low-voltage area ablation in persistent atrial fibrillation: sub-analysis of SUPPRESS-AF

**DOI:** 10.1093/ehjopen/oeag024

**Published:** 2026-02-20

**Authors:** Akihiro Sunaga, Yuki Matsuoka, Daisaku Nakatani, Katsuki Okada, Daisuke Sakamoto, Hideaki Hasegawa, Tetsuhisa Kitamura, Masaharu Masuda, Nobuaki Tanaka, Tetsuya Watanabe, Hitoshi Minamiguchi, Yasuyuki Egami, Takafumi Oka, Tomoko Minamisaka, Takashi Kanda, Masato Okada, Masato Kawasaki, Yasuhiro Matsuda, Koji Tanaka, Nobuhiko Makino, Hirota Kida, Shungo Hikoso, Tomoharu Dohi, Koichi Inoue, Yohei Sotomi, Yasushi Sakata, the OCVC-SUPPRESS-AF investigators, Masaharu Masuda, Takashi Kanda, Yasuhiro Matsuda, Hiroyuki Uematsu, Toshiaki Mano, Koichi Inoue, Tsuyoshi Mishima, Tatsuhisa Ozaki, Takuya Ohashi, Yasunori Ueda, Tetsuya Watanabe, Yoshio Furukawa, Masato Kawasaki, Mitsutoshi Asai, Takahisa Yamada, Nobuhiko Makino, Hitoshi Minamiguchi, Akio Hirata, Yoshiharu Higuchi, Yasuyuki Egami, Masamichi Yano, Yasuharu Matsunaga-Lee, Masami Nishino, Yasushi Sakata, Shungo Hikoso, Daisaku Nakatani, Hiroya Mizuno, Toshihiro Takeda, Takafumi Oka, Tomoaki Nakano, Kentaro Ozu, Takayuki Sekihara, Katsuki Okada, Tomoharu Dohi, Yohei Sotomi, Akihiro Sunaga, Hirota Kida, Bolrathanak Oeun, Taiki Sato, Yuki Matsuoka, Daisuke Sakamoto, Nobuaki Tanaka, Masato Okada, Koji Tanaka, Yuko Hirao, Katsuomi Iwakura, Tomoko Minamisaka, Shiro Hoshida

**Affiliations:** Department of Cardiovascular Medicine, Osaka University Graduate School of Medicine, 2-2 Yamadaoka, Suita 565-0871, Japan; Department of Cardiovascular Medicine, Osaka University Graduate School of Medicine, 2-2 Yamadaoka, Suita 565-0871, Japan; Department of Cardiovascular Medicine, Osaka University Graduate School of Medicine, 2-2 Yamadaoka, Suita 565-0871, Japan; Department of Cardiovascular Medicine, Osaka University Graduate School of Medicine, 2-2 Yamadaoka, Suita 565-0871, Japan; Department of Transformative System for Medical Information, Osaka University Graduate School of Medicine, 2-2 Yamadaoka, Suita 565-0871, Japan; Department of Cardiovascular Medicine, Osaka University Graduate School of Medicine, 2-2 Yamadaoka, Suita 565-0871, Japan; Department of Cardiovascular Medicine, Osaka University Graduate School of Medicine, 2-2 Yamadaoka, Suita 565-0871, Japan; Department of Social and Environmental Medicine, Osaka University Graduate School of Medicine, 2-2 Yamadaoka, Suita 565-0871 Japan; Cardiovascular Center, Kansai Rosai Hospital, 3-1-69 Inabaso, Amagasaki 660-0064, Japan; Cardiovascular Center, Sakurabashi Watanabe Advanced Healthcare Hospital, 4-3-51 Nakanoshima, Kita-ku, Osaka 530-0005, Japan; Division of Cardiology, Osaka General Medical Center, 3-1-56 Mandaihigashi, Sumiyoshi-ku, Osaka 558-8558, Japan; Cardiovascular Division, Osaka Keisatsu Hospital, 2-6-40 Karasugatsuji, Tennoji-ku, Osaka 543-0042, Japan; Division of Cardiology, Osaka Rosai Hospital, 1179-3 Nagasonetyo, Kita-ku, Sakai 591-8025, Japan; Department of Cardiovascular Medicine, Osaka University Graduate School of Medicine, 2-2 Yamadaoka, Suita 565-0871, Japan; Department of Cardiovascular Medicine, Yao Municipal Hospital, 1-3-1 Ryugetyo, Yao 581-0069, Japan; Cardiovascular Center, Kansai Rosai Hospital, 3-1-69 Inabaso, Amagasaki 660-0064, Japan; Cardiovascular Center, Sakurabashi Watanabe Advanced Healthcare Hospital, 4-3-51 Nakanoshima, Kita-ku, Osaka 530-0005, Japan; Division of Cardiology, Osaka General Medical Center, 3-1-56 Mandaihigashi, Sumiyoshi-ku, Osaka 558-8558, Japan; Cardiovascular Center, Kansai Rosai Hospital, 3-1-69 Inabaso, Amagasaki 660-0064, Japan; Cardiovascular Center, Sakurabashi Watanabe Advanced Healthcare Hospital, 4-3-51 Nakanoshima, Kita-ku, Osaka 530-0005, Japan; Cardiovascular Division, Osaka Keisatsu Hospital, 2-6-40 Karasugatsuji, Tennoji-ku, Osaka 543-0042, Japan; Department of Cardiovascular Medicine, Osaka University Graduate School of Medicine, 2-2 Yamadaoka, Suita 565-0871, Japan; Cardiovascular Medicine, Nara Medical University, 840 Shijyotyo, Kashihara 634-8522, Japan; Department of Cardiovascular Medicine, Osaka University Graduate School of Medicine, 2-2 Yamadaoka, Suita 565-0871, Japan; Cardiovascular Division, National Hospital Organization Osaka National Hospital, 2-1-14 Hoenzaka, Chuo-ku, Osaka 540-0006, Japan; Department of Cardiovascular Medicine, Osaka University Graduate School of Medicine, 2-2 Yamadaoka, Suita 565-0871, Japan; Department of Cardiovascular Medicine, Osaka University Graduate School of Medicine, 2-2 Yamadaoka, Suita 565-0871, Japan

**Keywords:** Atrial fibrillation, Low-voltage area, Substrate ablation, Homogenization, Win ratio, Hierarchical clinical outcomes

## Abstract

**Aims:**

In persistent atrial fibrillation (AF), low-voltage areas (LVAs) in the left atrium are considered arrhythmogenic. Although substrate ablation targeting LVAs may reduce AF recurrence, its effect on broader clinical outcomes remains unclear, and procedural risks must be considered. This study aims to compare hierarchical clinical outcomes between pulmonary vein isolation (PVI) alone and PVI plus LVA ablation in patients with persistent AF and LVAs using a win ratio analysis.

**Methods and results:**

This was a *post hoc* sub-analysis of the SUPPRESS-AF trial, including 341 patients with LVAs out of 1364 randomized. Patients received either PVI alone (*n* = 171) or PVI with LVA ablation (*n* = 170). Hierarchical outcomes were analysed in order of clinical importance: all-cause death, symptomatic stroke, AF recurrence, bleeding, and periprocedural complications. Win ratio analysis was used for comparison. Baseline characteristics were balanced between groups. The PVI plus LVA group had longer procedure times and higher energy delivery. The win ratio analysis showed no significant difference between groups (win ratio: 1.01, 95% confidence interval: 0.73–1.39, *P* = 0.940). The PVI-alone group had numerically fewer adverse events, while the LVA ablation group showed a numerical reduction in AF recurrence. Subgroup analyses showed consistent findings.

**Conclusion:**

In patients with persistent AF and LVAs, LVA ablation added to PVI did not improve hierarchical clinical outcomes and prolonged procedures. Routine use of current LVA ablation strategies is not supported, though targeted substrate modification may warrant further research.

**Registration:**

UMIN-CTR, https://www.umin.ac.jp/ctry. UMIN000035940.

What’s new?

- This study is the first to evaluate low-voltage area (LVA) ablation using a hierarchical win ratio approach, integrating outcomes beyond atrial fibrillation (AF) recurrence.
- In patients with persistent AF and LVAs, additional LVA ablation did not improve hierarchical clinical outcomes compared with pulmonary vein isolation (PVI) alone.
- Pulmonary vein isolation alone showed numerically fewer adverse events, including death, ischaemic stroke, bleeding, and complications, while PVI + LVA ablation was associated with fewer AF recurrences.
- Subgroup analyses across demographic and clinical factors revealed no differential benefit of LVA ablation.
- These findings suggest that extensive substrate modification may reduce AF recurrence but does not translate into better overall prognosis when considering clinically prioritized outcomes.

## Introduction

Ablation (ABL) therapy for atrial fibrillation (AF) is widely performed.^1,2^ In addition to suppressing AF recurrence, it offers several clinical benefits, including improved prognosis,^3^ reduced risk of stroke,^4^ and lowering bleeding risk due to the potential discontinuing anticoagulation therapy post-procedure.^5^

The recurrence rate of persistent AF is higher than that of paroxysmal AF,^6^ often necessitating substrate modification through ABL, which may involve extensive left atrial ablation.^7–10^ However, such extensive left atrial ABL raises concerns about the development of the stiff left atrium syndrome,^11^ which may increase the risk of embolism^12,13^ and heart failure.^14^

Since it has not been conclusively established that extensive ablation is superior to pulmonary vein isolation (PVI) alone in suppressing AF recurrence,^15^ aggressive substrate modification aimed at reducing recurrence may, paradoxically, worsen overall prognosis. Therefore, the effectiveness of ABL should be evaluated not only in terms of recurrence suppression but also in relation to comprehensive clinical outcomes, including mortality, thromboembolic events, and complications. Given that the clinical importance of each outcome differs, we believe that treatment strategies should be assessed using a hierarchical approach.^16^

The aim of this study was to compare the clinical efficacy of low-voltage area (LVA) ABL based on a hierarchy of outcomes, including mortality, embolic events, AF recurrence, bleeding events, and periprocedural complications using data from the SUPPRESS-AF randomized trial,^17,18^ which evaluated the recurrence suppression effect of LVA ABL in patients with persistent AF.

## Methods

### Study design and participants

This study was conducted as a *post hoc* sub-analysis of the SUPPRESS-AF trial, a prospective, investigator-initiated, multicentre, randomized, open-label study conducted at eight centres in Japan.

Inclusion criteria were patients scheduled to undergo their first catheter ABL for persistent AF. Key exclusion criteria included age < 20 years, left atrial diameter ≥ 55 mm, prior cardiac surgery, valvular AF, maintenance haemodialysis, contraindications to ablation or anticoagulant therapy, history of stroke or systemic embolism within the past 6 months, a treatable underlying cause of AF, pregnancy, or cases deemed unsuitable by the attending physician.

Patients with left atrial LVAs identified by voltage mapping were randomized in a 1:1 ratio to either receive additional LVA ABL following PVI (PVI + LVA-ABL group) or undergo PVI alone (PVI-alone group). Randomization was performed immediately after voltage mapping using a centrally concealed, computer-generated allocation system. To minimize imbalances across centres, randomization was stratified by participating hospital using the minimization method. Allocation results were delivered to each site via a secure online system.

**Figure 1:**
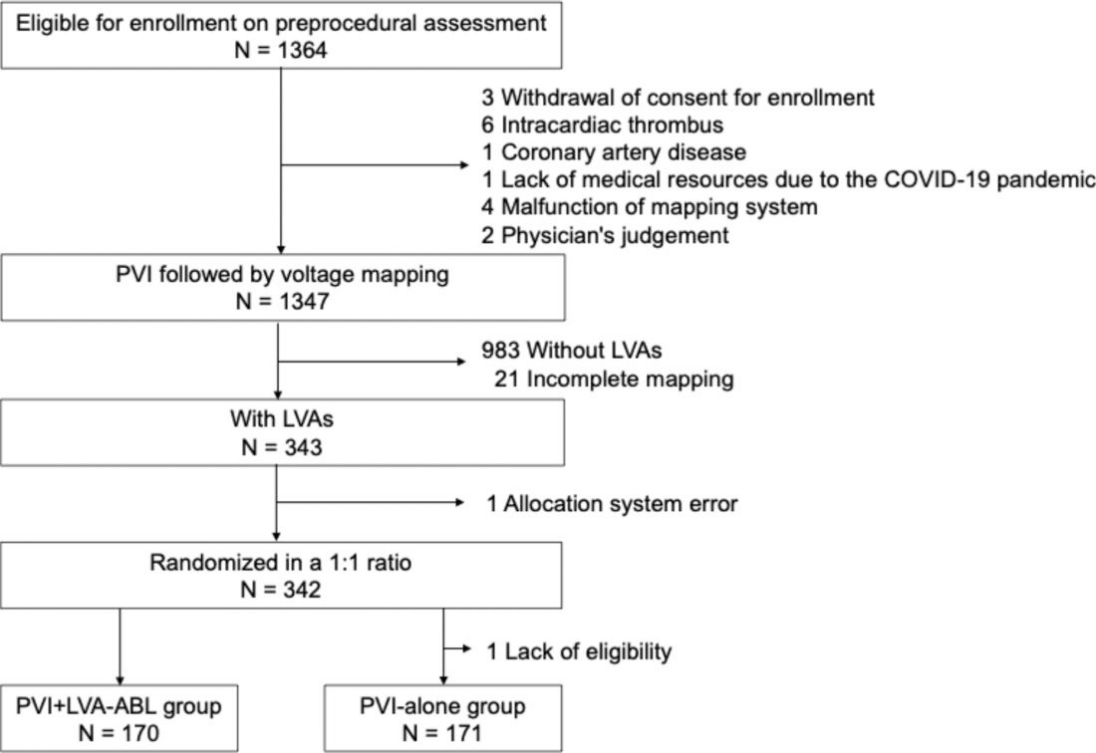
Patient flow. Of the 1364 patients enrolled in the SUPPRESS-AF study between June 2019 and August 2022, low-voltage areas were detected in 343 patients (25.5%). Among them, 342 patients were randomly assigned to either the PVI + LVA-ABL group or the PVI-alone group. For the final analysis, 170 patients in the PVI + LVA-ABL group and 171 patients in the PVI-alone group were included, excluding one patient who did not meet the eligibility criteria. AF, atrial fibrillation; COVID-19, coronavirus disease 2019; LVA, low-voltage area; PVI, pulmonary vein isolation; ABL, ablation.

Of the 1364 patients enrolled in the SUPPRESS-AF trial, 341 patients with identified LVA were included in this analysis, after excluding one patient due to an allocation system error and another who did not meet eligibility criteria. This sub-analysis compared hierarchical clinical outcomes between the PVI + LVA-ABL and PVI-alone groups (*Figure 1*).

The protocol for the SUPPRESS-AF trial was approved by the ethics committees of Osaka University and all participating institutions. Written informed consent was obtained from all participants before the ablation procedure. The trial and this sub-analysis were funded by Biosense Webster through the investigator-initiated study programme. The sponsor had no role in conducting the study beyond reviewing and approving the protocol. This study was conducted in accordance with the Declaration of Helsinki.

### Catheter ablation procedure

An electroanatomical mapping system (CARTO 3; Biosense Webster, Irvine, CA, USA) was used for catheter navigation, ABL guidance, and mapping. All patients underwent ipsilateral encircling PVI using an open-irrigated ABL catheter equipped with a contact force sensor (Thermocool SmartTouch SF®; Biosense Webster). Radiofrequency (RF) energy delivery was guided by the Visitag Surpoint® module (Biosense Webster), with target Visitag Surpoint values set at ≥425 for the anterior wall and ≥375 for the posterior wall, while maintaining an inter-lesion distance of ≤4 mm. The VISITAG module parameters were configured as follows: (i) catheter motion stability ≤ 2 mm, (ii) stability duration > 5 s, and (iii) contact force ≥ 5 g maintained for ≥25% of the time.

A waiting period of at least 20 min was observed following PVI, after which both entrance and exit blocks were confirmed for each ipsilateral pulmonary vein. If AF persisted at the completion of PVI, electrical cardioversion was performed.

Subsequently, left atrial voltage mapping was conducted during 100 ppm pacing from the high right atrium. Magnetic sensor-enabled multielectrode mapping catheters with 1 mm electrodes (Lasso Nav® or Pentaray®; Biosense Webster) were used. Mapping points were automatically collected using the Confidense Module® (Biosense Webster) to ensure complete coverage of all colour gaps in the voltage map. The module settings were cycle length filtering, ±30 ms; local activation time stability, 3 ms; position stability, 2 mm; density, 1 mm; tissue proximity indicator, off; and fill and colour interpolation threshold, 10 mm.

LVAs were defined as regions with a bipolar peak-to-peak voltage of <0.5 mV, with the scar threshold set at 0.05 mV. The total LVA area was manually measured using the CARTO system’s area measurement tool. The presence of an LVA was defined as the cumulative LVA ≥ 5 cm² in the left atrium.

In the PVI + LVA-ABL group, homogenization ABL was performed to cover all identified LVAs, except for dense scar regions (voltage < 0.05 mV), where ABL was omitted. Low-voltage areas located on the posterior wall could be isolated using roof and bottom lines. Each RF application was guided by a Visitag Surpoint threshold of ≥350, with an inter-lesion distance of <6 mm. ABL was avoided at locations where it might disrupt physiological conduction or cause damage to adjacent structures, such as the oesophagus.

After completing all ABL procedures, atrial burst stimulation and intravenous isoproterenol were administered to induce AF or atrial tachycardia (AT). Induced AT or non-pulmonary vein AF triggers were treated at the operator’s discretion. In the PVI-alone group, ABL within or adjacent to an LVA was permitted only when necessary to terminate AT or eliminate non-pulmonary vein AF triggers; prophylactic or deliberate LVA-targeting ABL was prohibited. Additionally, cavotricuspid isthmus ABL was permitted in cases of clinically evident tricuspid isthmus-dependent atrial flutter.

### Follow-up

Patients were followed for 12 months after the procedure and were required to visit the cardiology outpatient clinic at 6 and 12 months for clinical evaluation. Before each visit, a standard 12-lead electrocardiogram (ECG) was performed in the supine position, and a 24-h Holter ECG was recorded during routine daily activities.

In addition, from 6 to 12 months post-procedure, patients conducted twice-daily and symptom-triggered 30-s ECG recordings using a portable device (HCG 901 or HCG 801; Omron, Kyoto, Japan). This portable ECG device is approved by regulatory authorities, including those in the European Union and Japan, and has been used in multiple clinical studies.^19,20^

Recurrence of AF was defined as the occurrence of either (i) AF or atrial AT detected on scheduled or symptom-triggered ECG or (ii) AF/AT episodes lasting ≥30 s as documented on Holter ECG monitoring. Episodes occurring within the first 3 months post-procedure were not classified as recurrences, as this period was considered a blanking period. Antiarrhythmic drug use was not recommended for 3 months after the ABL procedure. The use of antiarrhythmic drugs beyond 3 months after the ABL was considered a recurrence of AF.

### Endpoints

The hierarchical clinical outcomes were defined as follows: (i) all-cause mortality, (ii) symptomatic cerebrovascular stroke, (iii) AF recurrence without the use of antiarrhythmic drag, (iv) bleeding events, and (v) perioperative complications.

Survival outcomes (death, stroke, AF recurrence, and bleeding events) were analysed as time-to-event endpoints, whereas periprocedural complications were evaluated as binary outcomes. Bleeding events were defined as major bleeding in the International Society of Thrombosis and Homostasis bleeding criteria^21^ or bleeding requiring hospitalization.

### Statistical analysis

Continuous variables are presented as mean ± standard deviation or median with interquartile range, as appropriate. Categorical variables are expressed as absolute numbers and percentages. All analyses were conducted based on the intention-to-treat principle. A two-sided *P* < 0.05 was considered statistically significant. Procedural characteristics were compared using the unpaired *t*-test for continuous variables and the χ^2^ test or Fisher’s exact test for categorical variables.

Win ratio analysis was performed to compare clinical outcomes between the treatment groups, incorporating a hierarchical structure of outcome importance. In this method, patients from the two groups are paired, and each pair is compared sequentially based on the predefined hierarchy of outcomes. Each pair is first assessed for the highest-priority outcome. If a difference is observed, the comparison is concluded. If not, the next outcome in the hierarchy is evaluated, and this process continues until a difference is found or all outcomes are assessed. The total number of wins and losses across all patient pairs is used to calculate the win ratio, which represents the ratio of favourable outcomes in the treatment group to those in the control group. The win ratio and its 95% confidence interval were estimated using the WinRatio package in R.^22^ All statistical analyses were conducted using R software (version 4.3.1; R Foundation for Statistical Computing, Vienna, Austria).

## Results

### Patient characteristics

Of the 1364 patients enrolled in the SUPPRESS-AF study between June 2019 and August 2022, 341 patients with identified LVAs were included in this analysis. One patient was excluded due to an allocation system error, and another did not meet the eligibility criteria. Among the 341 patients, 170 were assigned to the PVI + LVA-ABL group and 171 to the PVI-alone group (*Figure 1*).

Baseline characteristics were well balanced between the two groups, except for anticoagulants (*Table 1*). The mean age of the cohort was ∼74 years, and nearly half of the patients were female. Persistent AF lasting more than 1 year was observed in ∼21% of patients. The average left atrial diameter was ∼44 mm.

**Table 1:** Baseline characteristics

	PVI + LVA-ABL	PVI-alone	*P* value
	*n* = 170	*n* = 171	
Age, years	73.8 ± 6.8	74.7 ± 6.1	0.189
Female, *n* (%)	85 (50)	82 (48)	0.787
Body mass index, kg/m^2^	23.7 ± 4.1	23.7 ± 3.8	0.828
Heart rate, beats per min	81.4 ± 17.2	81.7 ± 17.1	0.863
Systolic blood pressure, mmHg	126 ± 18	126 ± 17	0.872
AF period, days	189 (83, 662)	180 (87, 602)	0.840
Duration of AF persistence, days	115 (63, 342)	110 (59, 290)	0.488
Long-standing persistent AF^a^, *n* (%)	38 (22)	32 (19)	0.485
Hypertension, *n* (%)	117 (68)	125 (74)	0.358
Diabetes mellitus, *n* (%)	42 (25)	35 (21)	0.420
Congestive heart failure, *n* (%)	54 (32)	49 (29)	0.663
NYHA functional Class II, *n*	35	33	
NYHA functional Class III, *n*	4	3	
NYHA functional Class IV, *n*	0	0	
Stroke, *n* (%)	15 (9)	19 (11)	0.600
Vascular disease, *n* (%)	3 (2)	4 (2)	>0.999
Myocardial infarction, *n* (%)	4 (2)	3 (2)	0.994
NT-pro BNP, pg/mL	1150 (737, 1874)	1008 (653, 1578)	0.130
eGFR, mL/min/1.73 m^2^	60.5 ± 17.0	60.5 ± 14.7	0.990
CHA_2_DS_2_-VASc score	3.4 ± 1.3	3.5 ± 1.5	0.381
Anticoagulants			0.039
Direct oral anticoagulants, *n (%)*	166 (98)	162 (95)	
Warfarin, *n (%)*	2 (1)	9 (5)	
Antiarrhythmic drugs, *n* (%)	9 (5)	13 (8)	0.518
Echocardiography			
Left atrial diameter, mm	44.1 ± 5.4	43.6 ± 5.5	0.401
Left ventricular ejection fraction, %	55.8 ± 10.7	57.4 ± 10.4	0.178

AF, atrial fibrillation; eGFR, estimated glomerular filtration rate; NT-proBNP, N-terminal prohormone of brain natriuretic peptide; NYHA, New York Heart Association.

^a^Long-standing persistent atrial fibrillation was defined as persistent atrial fibrillation lasting for >1 year.

### Procedural characteristics

*Table 2* shows procedural characteristics. The total procedure time, total RF application time, and total applied energy were significantly greater in the PVI + LVA-ABL group compared with the PVI-alone group. The first-pass isolation rates for both left- and right-sided PVIs were around 90% in both groups, with no significant difference between them. The mean LVA size was similar between the groups. The average RF time dedicated to LVA ablation was 648 s, with a median applied energy of 21.5 kJ. There was no significant difference between groups in the proportions of patients undergoing non-pulmonary vein AF trigger ablation or cavotricuspid isthmus ABL. ABL for regular AT was more frequent in the PVI + LVA-ABL group.

**Table 2:** Procedural characteristics

	PVI + LVA-ABL	PVI-alone	*P*
	*n* = 170	*n* = 171	
Total procedure time, min	192.1 ± 72.8	163.8 ± 59.1	<0.001
Total ablation time, s	2461 ± 916	1761 ± 575	<0.001
Total applied radiofrequency energy, kJ	86.2 ± 27.0	63.2 ± 19.8	<0.001
Deflectable sheath, *n* (%)	134 (79)	134 (78)	>0.999
Mapping catheter			
Circular catheter, *n* (%)	23 (14)	21(12)	0.749
Radiating catheter, *n* (%)	147 (87)	150 (88)	
Mapping points, *n*	1876 ± 761	1843 ± 772	0.689
Mapping time, min	18.5 ± 8.1	17.4 ± 7.0	0.163
LVA size, cm^2^	13.2 (8.5–21.5)	14.0 (8.7–24.3)	0.800
Left atrial surface area size, cm^2^	164.5 (136.8, 195.8)	158.2 (132.6, 185.0)	0.129
LVA ablation	170 (100)	0	
Applied energy, kJ	21.5 (13.2, 30.6)		
Ablation time, s	648 ± 357		
Complete homogenization, *n* (%)	133 (78)		
Pulmonary vein isolation, *n* (%)	170 (100)	171 (100)	
First-pass isolation (left side), *n* (%)	151 (89)	149 (87)	0.740
First-pass isolation (right side), *n* (%)	147 (87)	151 (88)	0.628
Non-pulmonary vein AF trigger ablation, *n* (%)	9 (5)	17 (10)	0.152
Superior vena cava, *n* (%)	3 (2)	5 (3)	0.723
Right atrium, *n* (%)	2 (1)	4 (2)	0.685
Left atrium, *n* (%)	5 (3)	7 (4)	0.770
Coronary sinus, *n* (%)	1 (1)	1 (1)	>0.999
Cavotricuspid isthmus ablation	43 (25)	40 (23)	0.706
For clinical AFL, *n* (%)	6 (4)	4 (2)	0.542
For induced AFL, *n* (%)	36 (21)	35 (21)	0.895
As empirical ablation, *n* (%)	1(1)	1 (1)	>0.999
Ablation of regular AT, *n* (%)	28 (17)	12 (7)	0.007
Perimitral AT, *n* (%)	6 (4)	3 (2)	0.336
Roof-dependent AT, *n* (%)	5 (14)	3 (19)	0.502
Other ATs, *n* (%)	19 (11)	10 (6)	0.084

AF, atrial fibrillation; AFL, atrial flutter; AT, atrial tachycardia; LVA, low-voltage area.

In this study, “other AT” in *Table 2* was defined as any regular AT other than perimitral or roof-dependent AT. In the PVI + LVA-ABL group, 27 distinct other ATs (tachycardia-level) were identified in 19 patients, whereas 12 distinct other ATs were identified in 10 patients in the PVI-alone group. The origin of each other AT is shown in Supplementary material online, *Table S1*.

### Endpoints

**Figure 2:**
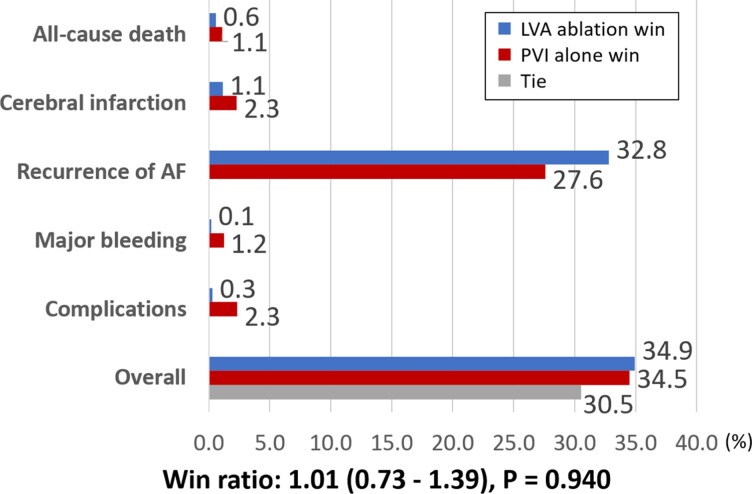
Win ratio for clinical endpoints. The hierarchical composite outcome, including all-cause death, stroke, atrial fibrillation recurrence of atrial, bleeding events, and periprocedural complications, was analysed using the win ratio. Each participant in the PVI + LVA-ABL group was compared to every participant in the PVI-alone group to determine the number of wins. The win ratio, calculated as the total number of wins in the PVI + LVA-ABL group divided by those in the PVI-alone group, is presented with its corresponding 95% confidence interval. LVA, low voltage area; PVI, pulmonary vein isolation; ABL, ablation.

In the hierarchical outcome analysis based on clinical importance—prioritized as all-cause death, cerebral infarction, AF recurrence, bleeding events, and periprocedural complications—no significant difference was observed between the two groups in the win ratio analysis. Numerically, the PVI-alone group showed favourable trends for all-cause death, cerebral infarction, bleeding events, and complications, whereas the PVI + LVA-ABL group demonstrated a favourable trend only for AF recurrence (*Figure 2*). At 6 and 12 months after ABL, oral anticoagulant use was lower in the PVI + LVA-ABL group than in the PVI-alone group (see Supplementary material online, *Table S2*).

**Figure 3:**
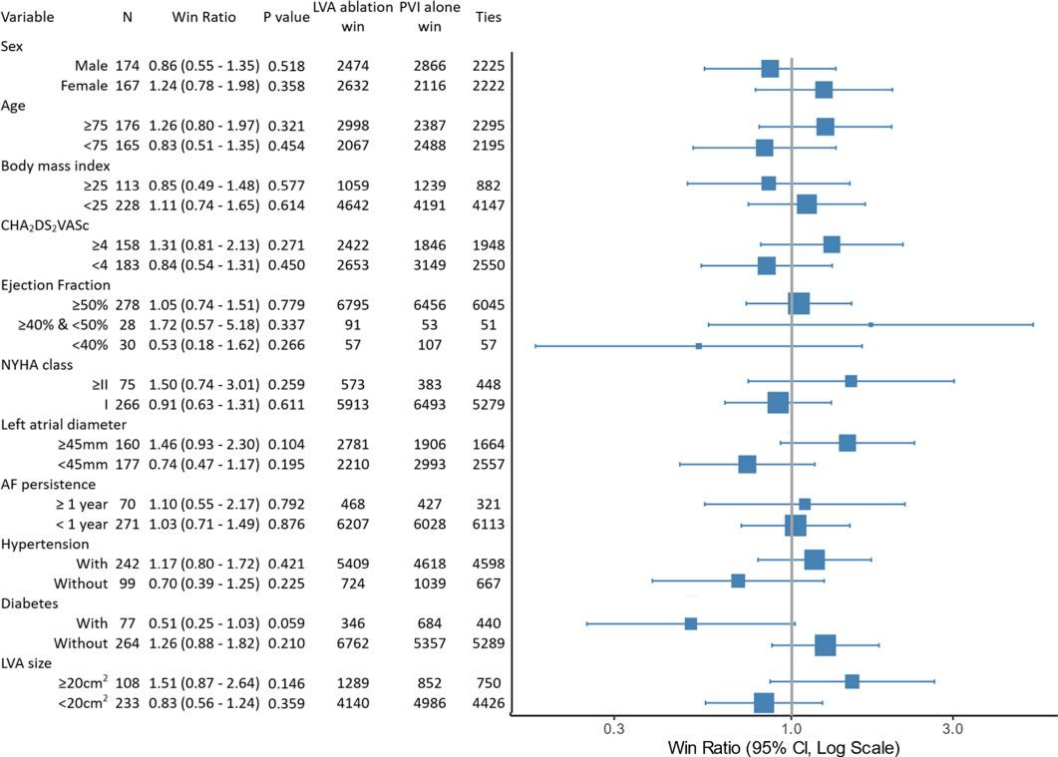
Subgroup analysis of win ratio for clinical endpoints. The win ratio for the hierarchical composite outcome (all-cause death, stroke, AF recurrence, bleeding events, and periprocedural complications) was analysed across prespecified subgroups. LVA, low voltage area; PVI, pulmonary vein isolation.

Subgroup analyses stratified by sex, age, body mass index, CHA₂DS₂-VASc score, left ventricular ejection fraction, New York Heart Association class, left atrial diameter, AF persistence, hypertension, diabetes, and LVA size revealed no significant differences in hierarchical outcomes between the two groups in any subgroups (*Figure 3*).

Details of each clinical outcome are presented in *Table 3*. The PVI-alone group showed numerically fewer events for death, ischaemic stroke, bleeding, and complications, whereas the PVI + LVA-ABL group had fewer AF recurrences. For ischaemic stroke, the PVI + LVA-ABL group had a higher number of events during the non-periprocedural period. Regarding complications, heart failure was more frequently observed in the PVI + LVA-ABL group.

**Table 3:** Details of outcomes

	PVI + LVA-ABL	PVI-alone	*P*
	*N* = 170	*N* = 171	
Death, *n* (%)	2 (1.2)	1 (0.6)	0.996
Malignancy, *n*	1	1	
Unknown, *n*	1	0	
Ischaemic stroke, *n* (%)	4 (2.4)	3 (1.8)	0.994
Within 1 week, *n*	0	1	
1 week–1 month, *n*	0	1	
1–12 months, *n*	4	1	
Recurrence of AF^a^, *n* (%)	65 (38.2)	81 (47.4)	0.111
Atrial fibrillation, *n*	39	58	
Atrial tachycardia, *n*	22	13	
Antiarrhythmic drug use, *n*	4	10	
Bleeding events (per patient), *n* (%)	6 (3.5)	2 (1.2)	0.279
In critical organ, *n*	2	1	
Fall in haemoglobin level of 2 mg/dL, *n*	2	0	
Blood transfusion, *n*	2	1	
Requiring hospitalization, *n*	5	1	
Complications (per patient), *n* (%)	11 (6.5)	4 (2.3)	0.110
Heart failure, *n*	5	1	
Ischaemic stroke, *n*	0	1	
Groin haematoma necessitating intervention, *n*	2	0	
Aspiration pneumonia, *n*	1	0	
Blood transfusion, *n*	1	1	
Coronary spasm, *n*	1	0	
Pericarditis, *n*	0	1	
Cardiac tamponade, *n*	0	1	
Oesophageal fistula, *n*	1	0	

AF, atrial fibrillation.

^a^The use of antiarrhythmic drugs beyond 3 months after the ablation was considered a recurrence of atrial fibrillation.

## Discussion

### Main findings

In this sub-analysis of the SUPPRESS-AF trial, we compared the clinical impact of additional LVA ABL with PVI alone in patients with persistent AF and documented left atrial LVAs. The addition of LVA ablation did not result in a significant improvement in hierarchical clinical outcomes—including all-cause death, stroke, AF recurrence, bleeding events, and periprocedural complications—compared with PVI alone.

### Win ratio analysis

In many previous randomized controlled trials, safety and efficacy have been assessed separately.^8,9^ While composite endpoints are occasionally employed,^3,23^ they typically do not allow for simultaneous evaluation of efficacy and safety, and events with varying clinical importance—such as death and heart failure hospitalization—are often weighted equally. Given the varying clinical importance of different outcomes, they should be appropriately weighted or considered in the analysis. Extensive left atrial ABL may lead to stiff left atrium (stiff LA) syndrome and result in adverse outcomes.

Therefore, in ABL strategies that involve additional extensive left atrial ABL, such as LVA ABL, it is particularly important to evaluate clinical outcomes in a hierarchical manner. To our knowledge, this is the first study to assess hierarchical outcomes using a win ratio analysis in patients with persistent AF and LVAs, comparing additional LVA ABL with PVI alone.

### Clinical trade-offs and future perspectives of low-voltage area ablation

Although no statistically significant differences were observed, numerical trends suggested that additional LVA ABL was associated with better AF/AT recurrence suppression. However, it tended to be inferior to PVI alone in terms of mortality, stroke, bleeding, and periprocedural complications.

The efficacy of ablation targeting LVA has not yielded consistent results across studies. The ERASE-AF trial, employing a strategy of isolating the LVA with linear ABL, has demonstrated its effectiveness.^8^ However, in the present study, we adopted a homogenization approach for the LVA using a Visitag Surpoint threshold of ≥350. The absence of a significant difference in efficacy may be due to the possibility that the ABL of the LVA was insufficient with the ABL technique used in this study.

It has been reported that even a very low residual potential of 0.04 mV, remaining after LVA homogenization, can serve as a substrate for iatrogenic AT.^24^ If a strategy that isolates the LVA using linear ablation is not employed, there may be a risk of incomplete elimination of arrhythmogenic substrates. In the present study, the insufficiency of ABL is suggested by the fact that the higher incidence of atrial tachycardia recurrence is observed in the PVI + LVA-ABL group.

If complete modification of the LVA had been achieved, recurrences due to atrial tachycardia might have been better suppressed, potentially resulting in superior efficacy in preventing AF recurrence in which AT recurrence was included in this trial. In terms of homogenization, further ABL technique such as pulsed field ABL may contribute to the complete elimination of arrhythmogenic substrate. LVA ABL remains a potentially effective strategy in terms of suppression of AF recurrence.

On the contrary, adverse events were more frequently observed in the LVA ablation group.^8,25,26^ These unfavourable outcomes may be attributable to factors, such as more extensive myocardial injury and longer procedural time. The use of alternative energy sources, such as pulsed field ablation—which causes less collateral damage, induces minimal inflammation,^27–29^ and prevents chronic atrial fibrotic changes^30^—may help mitigate these procedural risks.

In this study, the higher incidence of cerebral infarction in the chronic phase and heart failure in the periprocedural phase observed in the PVI + LVA-ABL group is likely attributable to extensive injury to the left atrium and prolonged procedure time. As long as additional ABL is performed, these issues—prolonged procedure time and atrial injury—are challenges that are likely to persist regardless of the ABL technique or energy source used. This is likely to be particularly important for AF patients with arrhythmogenic substrates, who are at higher risk of death, stroke, and heart failure compared to those without such substrates.^31^ Therefore, future studies should emphasize not only the effectiveness in preventing recurrence but also the importance of evaluating safety outcomes in parallel.

### Clinical implications

Based on the current findings, routine implementation of LVA ABL as performed in this study is not supported. If LVA ABL is to be pursued, careful attention should be paid to periprocedural complications, particularly heart failure. In addition, the potential for increased stroke risk during the chronic phase warrants close consideration. These concerns highlight the importance of evaluating the effectiveness of AF ABL not only by arrhythmia recurrence but also through broader, patient-centred outcomes that encompass cardiovascular events and overall clinical well-being. Further studies are warranted to identify optimal ABL techniques and energy sources that can improve such comprehensive outcomes in patients with persistent AF and LVAs.

### Limitation

Several limitations of this study warrant consideration. First, as this was a *post hoc* sub-analysis of the SUPPRESS-AF trial, the findings should be interpreted with caution. Second, because the study was conducted exclusively at institutions in Japan, the generalizability of the findings to other populations may be limited. Third, the number of high-priority clinical events, such as death and stroke, was relatively small, which may have limited the statistical power to detect meaningful differences between groups. Fourth, the follow-up period was limited to 12 months; therefore, long-term outcomes and late-onset complications could not be evaluated. Fifth, although recurrence was assessed using both Holter monitoring and handheld ECG devices, asymptomatic AF episodes may have gone undetected, potentially resulting in an underestimation of the true recurrence rate. Finally, although quality-of-life (QOL) data were not included, incorporating QOL into the hierarchy could have made the analysis more informative.

## Conclusions

In patients with persistent AF and LVAs, the addition of LVA ABL to PVI did not improve the hierarchical clinical outcomes, suggesting that routine implementation of LVA AVL using the current strategy may not be recommended.

## Supplementary Material

oeag024_Supplementary_Data

## Data Availability

The datasets from the present study are not publicly available due to concerns about patient confidentiality and proprietary considerations. Deidentified individual patient-level clinical data will be available on request for academic use with appropriate consideration of patient confidentiality.
