# Supplementary material for: Win ratio analysis of low-voltage area ablation in persistent atrial fibrillation: sub-analysis of SUPPRESS-AF
Source: Eur Heart J Open. 2026 Feb 20;6(1):oeag024. doi: 10.1093/ehjopen/oeag024 (PMC12961379; doi:10.1093/ehjopen/oeag024)
Supplement: oeag024_Supplementary_Data [file oeag024_supplementary_data.docx]

**Supplementary Table1. Origins of Other ATs during initial ablation**

|  | PVI+LVA-ABL | PVI-alone |
| --- | --- | --- |
| **Left atrium, n** | 8 | 6 |
| **Right atrium, n** | 12 | 4 |
| **Biatrial macroreentry, n** | 2 | 0 |
| **Coronary sinus, n** | 2 | 0 |
| **Atrioventricular nodal reentrant tachycardia, n** | 2 | 1 |
| **Undetermined, n** | 1 | 1 |

AT, atrial tachycardia; PVI, pulmonary vein isolation; LVA, low-voltage area; ABL, ablation.

**Supplementary Table2. Oral anticoagulation after ablation**

|  | PVI+LVA-ABL | PVI-alone | P value |
| --- | --- | --- | --- |
|  | n=170 | n=171 |  |
| **Anticoagulants at discharge** |  |  | 0.179 |
| *None, n (%)* | 0 (0) | 0 (0) |  |
| *Direct oral anticoagulants, n (%)* | 168 (99) | 164 (96) |  |
| *Warfarin, n (%)* | 2 (1) | 7 (4) |  |
| **Anticoagulants at 3 months** |  |  | 0.159 |
| *None, n (%)* | 10 (6) | 5 (3) |  |
| *Direct oral anticoagulants, n (%)* | 150 (93) | 152 (93) |  |
| *Warfarin, n (%)* | 2 (1) | 6 (4) |  |
| **Anticoagulants at 6 months** |  |  | 0.031 |
| *None, n (%)* | 21 (13) | 9 (6) |  |
| *Direct oral anticoagulants, n (%)* | 143 (86) | 150 (91) |  |
| *Warfarin, n (%)* | 2 (1) | 6 (4) |  |
| **Anticoagulants at 12 months** |  |  | 0.041 |
| *None, n (%)* | 32 (20) | 18 (11) |  |
| *Direct oral anticoagulants, n (%)* | 124 (79) | 135 (85) |  |
| *Warfarin, n (%)* | 2 (1) | 6 (4) |  |

PVI, pulmonary vein isolation; LVA, low-voltage area; ABL, ablation.
